# BRCA Mutations in Prostate Cancer: Prognostic and Predictive Implications

**DOI:** 10.1155/2020/4986365

**Published:** 2020-09-07

**Authors:** Carlo Messina, Carlo Cattrini, Davide Soldato, Giacomo Vallome, Orazio Caffo, Elena Castro, David Olmos, Francesco Boccardo, Elisa Zanardi

**Affiliations:** ^1^Department of Medical Oncology, Santa Chiara Hospital, 38122 Trento, Italy; ^2^Department of Internal Medicine and Medical Specialties (DIMI), University of Genoa, 16132 Genoa, Italy; ^3^Prostate Cancer Clinical Research Unit, Spanish National Cancer Research Centre (CNIO), Madrid 28029, Spain; ^4^Academic Unit of Medical Oncology, IRCCS Ospedale Policlinico San Martino, 16132 Genoa, Italy; ^5^Genitourinary Tumors Traslational Research Group, Biomedical Research Institute of Malaga and Hospital Universitario Virgen de la Victoria, 29010 Malaga, Spain

## Abstract

Despite chemotherapy and novel androgen-receptor signalling inhibitors (ARSi) have been approved during the last decades, metastatic castration-resistant prostate cancer (mCRPC) remains a lethal disease with poor clinical outcomes. Several studies found that germline or acquired DNA damage repair (DDR) defects affect a high percentage of mCRPC patients. Among DDR defects, BRCA mutations show relevant clinical implications. BRCA mutations are associated with adverse clinical features in primary tumors and with poor outcomes in patients with mCRPC. In addition, BRCA mutations predict good response to poly-ADP ribose polymerase (PARP) inhibitors, such as olaparib, rucaparib, and niraparib. However, concerns still remain on the role of extensive mutational testing in prostate cancer patients, given the implications for patients and for their progeny. The present comprehensive review attempts to provide an overview of BRCA mutations in prostate cancer, focusing on their prognostic and predictive roles.

## 1. Introduction

Prostate cancer (PCa) is the second most common neoplasm in men worldwide and the second leading cause of cancer deaths in Western countries [1]. In the USA, 165,000 new cases and 29,000 deaths are estimated annually due to PCa [2]. Despite a median overall survival (OS) of 42.1 months, the failure-free survival (FFS) was only 11.2 months in patients with metastatic hormone-sensitive PCa enrolled in the control arm of the STAMPEDE trial [3]. Moreover, PCa patients live most of their natural history of disease in the castration-resistant setting, and in the last decade, the approval of six novel treatments for the management of metastatic castration-resistant prostate cancers (mCRPC), spanning from chemotherapy agents (docetaxel and cabazitaxel), androgen-receptor signalling inhibitors (ARSi, i.e., enzalutamide and abiraterone), vaccines (sipuleucel-T), and bone-seeking radiopharmaceuticals (radium-223), has dramatically changed the management of mCRPC [4]. Despite meaningful advances in PCa care, the clinical outcome of mCRPC patients is still poor, and the median OS is unsatisfactory, ranging approximately between 18 and 36 months [4]. A better understanding of the molecular characterization of mCRPC patients is an urgent medical need in order to better define diagnosis and prognosis and to deliver appropriate treatment. PCa is one of the most heritable human tumors [5]; the integrative analysis of advanced prostate cancer has revealed that approximately 90% of mCRPC patients harbor clinically actionable germline and somatic alterations [6]. In this scenario, DNA damage repair defects (DDR) represent 25% of these alterations, with BRCA2 mutations representing the most frequent event [6–8]. Inherited mutations in BRCA genes are associated with an increased risk of developing breast, ovarian, prostate, and other cancers [7, 8]. DDR genes are involved in the mechanisms of genomic stability, repairing DNA aberrations during the cell cycle, ensuring a correct mitotic cell division, and distribution of the genomic material to the daughter cells [9]. In order to solve threats generated by DNA damage, cells have developed several processes of DNA-damage response that detect DNA lesions, signal their presence, and promote the repair [10]. If the extent of DNA damage is beyond repair capacity, alternative signalling pathways lead to apoptosis of potentially dangerous mutated cells [11]. Several DNA repair pathways are involved to cope with different DNA lesions, and they usually occur by a common general program [12]. BRCA1/2 is a protein encoded by the major oncogene responsible for the susceptibility of breast and ovarian cancers and plays a key role in the system of the homologous recombination (HR), working simultaneously with several enzymes to protect the genome from double DNA strand breaks [13]. BRCA2 mutations are a strong negative prognostic factor associated with short metastasis-free survival (MFS) and cancer-specific survival (CSS) in patients with mCRPC [14]. Moreover, BRCA mutations can predict response to poly-ADP ribose polymerase (PARP) inhibitors and to platinum salts [15, 16]. The following attempts to provide a comprehensive review of the literature on BRCA mutations in patients suffering from PCa, highlighting their prevalence and prognostic and predictive role, as well as their implications for hereditary cancer and genetic counselling.

### 1.1. Prevalence of BRCA Mutations in Prostate Cancers

The incidence of germline mutation in DDR genes among men with metastatic PCa varies between 11% and 33%, and it is significantly higher compared to the incidence in men with localized PCa [15, 17]. In a landmark study, Pritchard and colleagues showed that 11% of 692 patients with metastatic PCa harbored inherited mutations in 16 DDR genes [17]. The most frequent aberration was BRCA2 (5.3%) followed by ATM (1.6%), CHEK2 (1.9%), BRCA1 (0.9%), and RAD51 (0.4%). Mutation frequency did not differ based on PCa family history or age at diagnosis [17]. In a multi-institutional integrative clinical sequencing analysis, 23% of 150 mCRPC biopsies were found to be positive for DDR aberrations. BRCA2 was mutated in 13% of samples followed by ATM (7.3%), MSH2 (2%), BRCA1, FANCA, MLH1, and RAD51 (0.3%) [15].

Several studies showed a different genomic landscape in mCRPC compared to localized PCa [6, 18]. In a large retrospective study, Robinson et al. analyzed 680 primary tumors and 333 mCRPC biopsies [6]. The authors identified germline and/or somatic DDR defects in 10% of primary tumors and 27% of metastatic samples. The different molecular profile between localized PCa and metastatic lesions might be a direct consequence of tumor evolution under the selective pressure of ARSi or chemotherapy. However, small subpopulations of variant clones might be already present in primary tumors and might expand during the development of metastatic disease.

In this regard, Mateo and colleagues profiled 470 treatment-naïve PCa biopsies from patients who developed lethal mCRPC; of these, 61 patients had matched samples of primary tumors and metastatic lesions [19]. DDR gene aberrations (BRCA2 7%; CDK12 5%; and ATM 4%), TP53 (27%), and PTEN (12%) were commonly detected. Interestingly, while AR, TP53, and RB1 mutations were more commonly found in mCRPC lesions compared to primary tumors, DDR mutations had similar prevalence in primary and mCRPC settings [19]. These findings suggested that the use of prostate biopsy might be useful to profile patients for DDR mutations, avoiding rebiopsies of metastatic lesions that are potentially dangerous and time-consuming. Moreover, these data supported the testing for DDR defects in earlier stages of PCa as many of these alterations are already present during the initial phases of PCa development. However, given the limits of the study by Mateo and colleagues, further studies are needed. In fact, the retrospective design of this study did not take into account different treatments received in the mCRPC setting and heterogeneity in primary tumors that might have resulted in different profile between primary and mCRPC lesions.

### 1.2. Clinical Implications of BRCA Mutations in Prostate Cancers

PCa is a clinically heterogeneous disease. Patients commonly show variable responses to treatments that result in different clinical outcomes. This clinical variability may reflect molecular heterogeneity. Therefore, molecular profiling could have a meaningful translational relevance, allowing to distinguish PCa with indolent behaviour from those with a lethal course. Several studies explored the prognostic role of BRCA mutations in localized PCa and in mCRPC patients treated with standard therapies [20]. In a large retrospective study, BRCA1/2 mutations correlated with higher Gleason score, nodal involvement, metastatic disease at diagnosis, and T3/4 stage [14]. Moreover, BRCA2 was an independent prognostic factor that was associated with poorer outcomes. In localized PCa, the 5-year CSS and MFS were significantly shorter in BRCA2 carriers than in noncarriers (82% vs. 96%; 77% vs. 93%, respectively) [14]. Given conflicting results reported in retrospective studies, it is currently uncertain whether BRCA2 mutation may affect the clinical outcome of mCRPC patients treated with standard treatments [21, 22]. Annala and colleagues retrospectively analyzed 319 charts of mCRPC patients, including 22 germline DDR (gDDR) carriers (16 BRCA2-mutated). Interestingly, gDDR carriers had a significant shorter progression-free survival (PFS) than noncarriers (3.3 vs. 6.2 months, *p*=0.01) when treated with first-line ARSi [21].

Antonarakis et al. evaluated the clinical significance of gDDR mutations in 172 mCRPC receiving first-line ARSi. Notably, in contrast to what was reported by Annala et al. [21], ATM-BRCA1/2 carriers had a trend towards longer PFS than noncarriers (15 vs. 10.8 months, *p*=0.090) [23]. Conversely, Mateo et al. found no difference in PFS on first-line ARSi (8.3 months in both groups) between gDDR carriers (*n* = 330) and noncarriers (*n* = 60) [22].

PROREPAIR-B was the first prospective trial designed to elucidate the prognostic impact of BRCA1/2, ATM, and PALB2 on CSS of mCRPC patients. All patients enrolled in this trial have not been treated with platinum or PARP inhibitors. Although the study failed to reach the primary endpoint of improved CSS between gDDR carriers (*n* = 68) and noncarriers (*n* = 351) (23.3 vs. 33.2 months; *p*=0.264), germline BRCA2 mutation (gBRCA2) was confirmed to be an independent prognostic factor that negatively affected CSS (17.4 months in gBRCA2 vs. 33.2 months in nonmutated patients; *p*=0.027) [24]. In a non-preplanned subgroup analysis of PROREPAIR-B, gBRCA2 mutation was also predicted for shorter CSS in mCRPC patients treated with the sequence docetaxel-ARSi compared to noncarriers (median 28.4 vs. 10.7 months, *p*=0.0003) [24]. In contrast, CSS of gBRCA2 carriers did not differ from that of noncarriers in patients treated with the sequence ARSi-docetaxel (31.2 vs. 24 months, *p*=0.901) [24]. This finding suggests that the choice of first-line therapy may affect the outcome of gBRCA2 patients, and these results may explain the aforementioned conflicting results from the three retrospective series [21, 22]. The multicenter and ambispective BRCA2MEN study is currently planned to validate the role of BRCA2 as a predictive biomarker to select the first-line therapy (ARSi vs. taxane) in patients with mCRPC.

### 1.3. Targeting BRCA Mutations in Prostate Cancer

#### 1.3.1. Platinum Agents

Platinum-based chemotherapy, alkylating DNA, induces genomic strand breaks that may be translated in a synthetic lethality in tumor cells with DDR mutation. Carboplatin is a standard treatment for BRCA1/2 patients in breast [25, 26]. and ovarian cancer [27]. Satraplatin provided a significant reduction in the risk of progression or death (HR 0.67; 95% CI, 0.57 to 0.77; *p* < 0.001) in a randomized phase 3 trial that enrolled unselected mCRPC patients who had progressed to prior taxane [28]. However, this benefit did not translate in OS advantage compared to placebo (HR = 0.98; 95% CI, 0.84 to 1.15; *p*=0.80).

Retrospective series and case reports also described the potential efficacy of platinum-based chemotherapy in mCRPC patients harboring gBRCA2 mutations [29, 30]. A retrospective study carried out at Dana-Farber Institute assessed the activity of carboplatin AUC 3–5 and docetaxel 60–75 mg/mq in 141 mCRPC patients who were previously progressed to standard therapies [16]. The combo significantly improved the rate of PSA decline in 6 out of 8 *BRCA2* carriers compared to 23 out of 133 noncarriers (*p*=0.001), and improved OS was also observed (18.9 in *BRCA2* carriers vs. 9.5 months in noncarriers) [16].

Several ongoing trials are evaluating the efficacy of platinum-based chemotherapy in mCRPC patients selected for DDR mutations [31–33].

#### 1.3.2. PARP Inhibitors

DDR defects cause the accumulation of genomic mutations in cancer cells, eventually leading to their proliferation, immortalization, and acquisition of an aggressive phenotype [34].

In vitro models showed that BRCA1- and BRCA2-defective cells are sensitive to PARP inhibitors, whereas BRCA1- and BRCA2-proficient cells are resistant [34].

**Figure 1:**
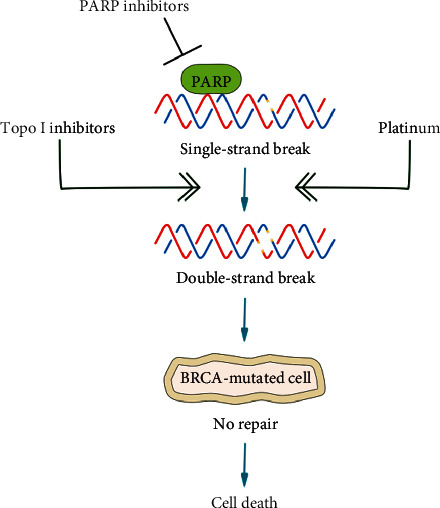
Mechanism of action of PARPi, platinum salts, and topoisomerase I inhibitors in the BRCA-mutated cell.

ADP-ribosylation is involved in several cellular processes, including cell growth and differentiation, apoptosis, and transcriptional regulation. However, ADP-ribosylation has a significant role in DNA repair and genome stability, promoting double-strand break repair via homologous recombination [35]. The blockade of PARP1 through the use of PARP inhibitors or alkylating agents causes accumulation of DNA damages in DDR-defective tumor cells, resulting in a synthetic lethality (Figure 1) [36]. Several PARP inhibitors have been developed and are under investigation in clinical research for mCRPC patients (see Table 1) [37]. Olaparib was the first PARP inhibitor that showed significant activity in patients with mCRPC who had progressed to standard treatments. In a phase II trial, 50 heavily pretreated, molecularly unselected, mCRPC patients received olaparib 400 mg twice a day until progression or unacceptable toxicities [15]. The primary composite endpoint was the objective response rate (ORR), defined according to RECIST 1.1 criteria or as a reduction of at least 50% in PSA levels or a confirmed reduction in the circulating tumor-cell count from 5 or more cells to less than 5 cells per 7.5 ml of blood [15]. The prevalence of gDDR alterations was 33%. In the whole population, 16 out of 49 evaluable patients had a response (33%; 95% CI, 20 to 48). Among patients with gDDR, 88% had a response to olaparib [15]. Moreover, olaparib significantly improved PFS (median 9.8 vs. 2.7 months; *p* < 0.001) and OS (median 13.8 months vs. 7.5 months *p*=0.05) of gDDR-mutated mCRPC patients compared to biomarker-negative patients [15]. In the randomized phase II TOPARP-B trial, 92 heavily pretreated mCRPC patients, selected for the presence of gDDR mutations, were randomized 1 : 1 to receive olaparib 400 mg twice daily or olaparib 300 mg twice daily [38]. The primary endpoint was defined as the presence of one of the following outcomes: radiological ORR assessed by RECIST 1.1 criteria, PSA response ≥50%, or circulating tumor-cell count conversion (from ≥5 cells per 7.5 mL blood at baseline to <5 cells per 7.5 mL blood). The primary endpoint was met. Composite response was achieved in 25 out of 46 patients receiving olaparib 400 mg (54.3%; 95% CI, 39.0–69.1) and in 18 out of 46 patients enrolled in olaparib 300 mg arm (39.1%; 25.1–54.6). The composite response was lower in patients treated with olaparib 300 mg, not reaching the prespecified criteria for success. However, almost 30% of patients treated with a higher dose of olaparib discontinued the treatment or reduced the schedule due to the development of grade 3-4 adverse events. Moreover, this trial showed that BRCA2-mutated patients had the greatest benefit from olaparib compared to those harboring CDK12 or ATM mutations. This trial suggested that the type of DDR mutation and olaparib dose had predictive implications. However, the type of mutation was not a stratification criterion for randomization; therefore, an allocation bias might have affected the results. The olaparib 300 mg arm was enriched of CDK12 patients, and this unbalance may have caused a lower benefit in this group of patients [38].

The role of DDR defects in predicting response to PARP inhibitors was more consistently demonstrated in the phase III PROFOUND trial, which randomized 387 mCRPC patients who were progressing to prior ARSi. Patients were allocated in two cohorts based on the presence of specific DDR defects (cohort A including BRCA1/2 or ATM and cohort B including other DDR defects). Olaparib 300 mg twice daily and second-line ARSi were administered in a 2 : 1 ratio [39]. The primary endpoint was radiological PFS (rPFS). Patients in cohort A treated with olaparib reported a median rPFS of 7.4 months compared to 3.55 months in those in the same cohort treated with ARSi (HR 0.34 (95% CI, 0.25–0.47), *p* < 0.0001). The PFS benefit was consistent throughout all subgroups within the prespecified subgroup analysis. Similar to that observed in the TOPARP-B trial [38], BRCA2-mutant patients had a better benefit from olaparib than patients harboring CDK12 or ATM mutations. The interim OS analysis also favored the olaparib arm (HR 0.64, 0.43–0.97), despite more than 80% of patients in the control group did crossover after disease progression. The ORR was 33% and 2.3% for experimental and control groups, respectively. Based on these findings, the US Food and Drug Administration (FDA) granted approval for olaparib in mCRPC patients with germline or somatic deleterious homologous recombination repair gene mutations who had progressed to prior ARSi on May 19, 2020.

The predictive value of DDR mutations was also confirmed in the preliminary findings from two phase II trials, TRITON-2 [40, 41] and GALAHAD [42], which investigated the activity of two other PARP inhibitors in patients with DDR-deficient mCRPC. In TRITON-2, mCRPC patients who had previously progressed to at least one ARSi and a taxane-based chemotherapy were screened for germline or somatic alterations in DDR genes. A total of 190 patients were treated with rucaparib 600 mg twice daily; the vast majority (98 pts) had BRCA1/2 alterations; and the remaining patients had alterations in ATM (57 pts), CDK12 (14), CHECK2 (7), and other genes (14 patients). ORR was 43.9% for patients with BRCA alterations, 9.5% for ATM, and 0% for the others. A similar pattern was observed for PSA response [41, 42]. On the basis of the preliminary results of the TRITON-2 trial, FDA announced on 15 May 2020 the accelerated approval of rucaparib for BRCA1/2 mCRPC patients progressing to prior ARSi or taxane.

In the GALAHAD trial, 165 patients with mCRPC and DDR defects progressing to at least one prior ARSi and taxane-based chemotherapy received niraparib 300 mg once daily. DDR positivity was defined by biallelic alterations in BRCA1/2, ATM, CHECK2, and other genes identified in plasma or tissue. ORR was the primary endpoint of the study. Patients who carried biallelic BRCA mutations achieved higher ORR (41% vs. 9%) and rPFS (8.2 months vs. 5.3) compared to those who did not harbor BRCA alterations [42]. It should be highlighted that the PROFOUND and the TRITON-2 trials evaluated mono- and biallelic alterations in DDR genes in tumor tissue and tumor tissue or plasma, respectively. Conversely, the GALAHAD trial required biallelic alterations in plasma samples to confirm eligibility. It is currently unknown whether the type and origin of BRCA mutations (germline vs. somatic and monoallelic vs. biallelic) could affect the response to treatment with PARP inhibitors.

### 1.4. Relevance of Germline Testing and Genetic Counselling

The high prevalence of DDR mutations and the clinical implications for their prognostic and predictive role have progressively led the international guidelines to implement recommendations for genetic and germline testing. The Philadelphia consensus conference recommends to test all patients with metastatic PCa, both in hormone-sensitive and castration-resistant settings, and in all patients with a significant family history of PCa or of tumors in the hereditary breast and ovarian cancer (HBOC) syndrome or Lynch syndrome spectrum. In metastatic PCa, both germline testing and somatic testing can be performed, and large gene panels can be used; however, the test should prioritize genes with more relevant clinical implications such as BRCA2, BRCA1, and mismatch repair (MMR). Furthermore, when somatic mutations are identified in BRCA2 or BRCA1, germline evaluation should also be performed due to the implications for all related family members. For patients with nonmetastatic PCa, the Philadelphia consensus suggests to use reflex testing, which consists of initial testing of priority genes followed by expanded testing, with a particular focus on BRCA2 results to personalize the strategies of active surveillance [43]. The US National Comprehensive Cancer Network (NCCN) guidelines recommend genetic testing (somatic and/or germline) for patients with high, very-risk, regional, and metastatic PCa or with a significant family history for cancer [44]. The recently published European Society for Medical Oncology (ESMO) guidelines recommend germline screening for all patients with mPCa and to consider genetic testing in patients with localized PCa and a family history suggestive for hereditary cancer predisposition [45]. Multidisciplinary discussion and integration with genetic services are fundamental to decide when and whether a genetic test should be performed and to select the appropriate therapeutic and diagnostic strategies. The IMPACT study is evaluating a screening strategy in men with gBRCA1/2 in order to define how to manage the population at a higher risk of PCa development in the presence of the BRCA2 mutation [46]. Annual prostate-specific antigen (PSA) tests and a biopsy for PSA >3 ng/ml are performed. Preliminary results revealed a higher incidence of PCa in gBRCA2 mutation carriers (3.3% vs. 2.6% in gBRCA1 mutation carriers, <2% for controls). Final results are awaited to be aware of the optimal screening strategies for this population.

## 2. Conclusions

Despite the development of several treatment options for mCRPC patients, metastatic PCa remains a lethal disease with poor prognosis [4]. Molecular characterization of mCRPC patients should be routinely integrated into the clinics in order to select patients who are more likely to respond to targeted agents and to minimize toxicities from unnecessary therapies. Furthermore, the emerging role of BRCA2 underlines the growing importance of genetic counselling and the multidisciplinary approach in the management of PCa patients. Recent evidence highlights that gBRCA2 is an independent prognostic factor associated with shorter CSS in mCRPC patients, and the type of first-line treatment might affect the outcome of gBRCA2 carriers [24]. Moreover, it has been demonstrated that gBRCA2 is a strong predictor of response to PARP inhibitors. The role of PARP inhibitors in non-BRCA DDR mCRPC patients remains less clear, and further studies should investigate this specific issue.

## Figures and Tables

**Table 1:** Ongoing clinical trials assessing the role of PARPi in mCRPC.

Clinical trial	Phase	Study drug	Strategy	Primary endpoint
NCT02861573	I	Olaparib	Pembrolizumab + olaparib in postdocetaxel setting	RR (PSA50)
NCT03874884	I	Olaparib	Olaparib + 177Lu-PSMA in mCRPC	DLTs, MTD, RP2D
NCT03205176	I	Olaparib	Olaparib ± AZD5153 (BRD4/BET bromodomain inhibitor) in mCRPC	DLT
NCT02484404	I/II	Olaparib	Olaparib ± ceridanib ± MEDI4736 (anti-PD-1) in mCRPC	RP2D, AE
NCT03317392	I/II	Olaparib	Ra223 ± olaparib in mCRPC patients with bone metastases	MTD, rPFS
NCT03787680	II	Olaparib	Olaparib + ATR inhibitor (AZD6738) in second-line setting	RR
NCT03012321	II	Olaparib	Olaparib ± abiraterone/prednisone in first-line setting	PFS
NCT03434158	II	Olaparib	Olaparib for patients who are responding after docetaxel chemotherapy	rPFS
NCT03263650	II	Olaparib	Olaparib for patients who are responding after cabazitaxel plus carboplatin	PFS
NCT03516812	II	Olaparib	Olaparib + testosterone enanthate in postabiraterone/enzalutamide setting	RR (PSA50)
NCT02893917	II	Olaparib	Olaparib ± cediranib in second-line setting	rPFS
NCT03732820	III	Olaparib	Abiraterone/prednisone ± olaparib in first-line setting	rPFS
NCT03834519	III	Olaparib	Olaparib plus pembrolizumab versus abiraterone acetate or enzalutamide after chemotherapy and ARSi	OS and PFS
NCT03076203	I	Niraparib	Niraparib + radium-223	MTD
NCT03431350	I/II	Niraparib	Niraparib + abiraterone/prednisone or JNJ-63723283 in post-ARSi setting	AE, ORR
NCT02854436	II	Niraparib	Niraparib in postdocetaxel and post-ARSi settings	ORR
NCT03748641	III	Niraparib	Abiraterone/prednisone ± niraparib in first-line setting	rPFS
NCT04179396	I	Rucaparib	Rucaparib + abiraterone or enzalutamide in mCRPC	PK, AE
NCT03840200	I	Rucaparib	Rucaparib + ipatasertib in mCRPC after ARSi	AE, DLTs, PSA response
NCT04253262	I/II	Rucaparib	Rucaparib + copanlisib (PI3K inhibitor) in mCRPC progressing after ARSi	MTD, ORR
NCT03840200	I/II	Rucaparib	Rucaparib + ipatasertib after ARSi	AE, DLT, RR (PSA)
NCT03572478	I/II	Rucaparib	Rucaparib vs. rucaparib + nivolumab vs. nivolumab	DLT
NCT02952534	II	Rucaparib	Rucaparib in postdocetaxel and post-ARSi settings	ORR
NCT03338790	II	Rucaparib	Nivolumab + rucaparib or docetaxel or enzalutamide	ORR
NCT03442556	II	Rucaparib	Rucaparib for patients who are responding after docetaxel plus carboplatin	rPFS
NCT02975934	III	Rucaparib	Rucaparib vs. abiraterone/enzalutamide/docetaxel in second-line setting	rPFS
NCT04019327	I/II	Talazoparib	Talazoparib + temozolomide in mCRPC without DNA damage repair mutation after at least one ARSi	AE, ORR
NCT04052204	I/II	Talazoparib	Talazoparib + avelumab + bempegaldesleukin in mCRPC	DLT, ORR
NCT03330405	II	Talazoparib	Avelumab plus talazoparib in advanced solid tumors	DLT, ORR
NCT03148795	II	Talazoparib	Talazoparib in postdocetaxel and postabiraterone/enzalutamide settings	ORR
NCT03395197	III	Talazoparib	Enzalutamide ± talazaparib in first-line setting	rPFS
NCT04182516	I	NMS-03305293	NMS-03305293 (PARP inhibitor) in mCRPC	First cycle DLTs

RR: response rate; PSA50: decline in PSA level ≥50% than baseline; MTD: maximum tolerated dose; rPFS: radiological progression-free survival; PFS: progression-free survival; OS: overall survival; AE: adverse events; ORR: objective response rate; DLT: dose-limiting toxicities; MTD: maximum tolerated dose; RP2D: recommended phase II dose; and PK: pharmacokinetic.
